# Factors associated with health professionals decision to initiate paediatric advance care planning: A systematic integrative review

**DOI:** 10.1177/0269216320983197

**Published:** 2020-12-29

**Authors:** Karen Carr, Felicity Hasson, Sonja McIlfatrick, Julia Downing

**Affiliations:** 1 Institute of Nursing and Health Research, Ulster University, Newtownabbey, UK; 2 International Children’s Palliative Care Network, UK & Makerere University, Kampala, Uganda

**Keywords:** Child, infant, adolescent, paediatric, advance care planning, palliative care, terminal care, decision making

## Abstract

**Background:**

Advance care planning for children with palliative care needs is an emotionally, legally and complex aspect of care, advocated as beneficial to children, families and health professionals. Evidence suggests healthcare professionals often avoid or delay initiation. An overview of evidence on the factors that influence and impact on the health care professional’s initiation of paediatric advance care planning process is lacking.

**Aim:**

To review and synthesise evidence on the factors associated with health care professional’s decision to initiate paediatric advance care planning.

**Design:**

Systematic integrative review using constant comparison method.

**Data Sources:**

Electronic databases (CINAHL, PubMed, PsycINFO, Ovid MEDLINE, EMBASE, Web of Science and Cochrane) using MeSH terms and word searches in Oct 2019. No limit set on year of publication or country. Grey literature searches were also completed.

**Results:**

The search yielded 4153 citations from which 90 full text articles were reviewed. Twenty-one met inclusion criteria consisting of quantitative (*n* = 8), qualitative (*n* = 6) and theoretical (*n* = 7) studies.

Findings revealed overarching and interrelated themes ‘*The timing of initiation’, ‘What makes an initiator, ‘Professionals’ perceptions’ and ‘Prerequisites to initiation’*.

**Conclusions:**

This review provides insights into the complexities and factors surrounding the initiation of advance care planning in paediatric practice. Uncertainty regarding prognosis, responsibility and unpredictable parental reactions result in inconsistent practice. Future research is required to inform intervention to assist health care professionals when initiating paediatric advance care planning conversations.

**What is already known about the topic?**

- Advance care planning in paediatrics is advocated however uptake remains low.
- Evidence to date is from adult populations and questions exist around transferability to a paediatric population.
- Delays in the initiation of advance care planning for this population result in discussions taking place at times of crises, perhaps when death is imminent which results emotionally charged discussions occurring.

**What this paper adds?**

- Initiation of advance care planning in paediatrics is influenced by an array of personal, social, cultural and organisational factors.
- It outlines important factors to consider when initiating paediatric advance care planning conversations with parents – such as developing a rapport, professional knowledge of paediatric advance care planning, educating the parent and approval to talk on the topic.

**Implications for practice, theory or policy**

- Initiation must happen as soon as opportune following recognition of a life limiting illness and should be rooted in the knowledge that paediatric advance care planning encompasses wishes whilst living as well as future planning and decision making and should not be focused solely on documenting restrictions to treatment and end of life plans.
- Professionals must be aware of the complexities of initiation but must also recognise that these should not act as a barrier to ensuring meaningful conversations occur.
- The use of a behaviour change theory in further research may provide evidence and on aspects of behaviour which could be adapted or changed to reduce the delay and avoidance behaviour evident in current practice.
- A standardised approach supported by education, guidelines and clinical tools is required to ensure paediatric advance care planning is initiated as a process and not seen as an anxiety evoking ‘one time’ conversation.

## Background

Globally, end of life planning, commonly referred to as advance care planning, is advocated in policy for both adult^1,2^ and paediatric palliative care.^3–6^ Advance care planning is a term used to describe ongoing conversations, between a person and family members and health professionals about future care and preferences. In paediatrics, advance care planning is supported by parents^7–9^ and professionals^10^ and linked to positive outcomes such as enhanced quality of life, care, satisfaction and reduced distress for patients and families.^10–12^ However, whilst it is recommended that paediatric advance care planning discussions start at the point of diagnosis or recognition of a life-limiting or life-threatening condition there are no formal national or international guidelines on how, when and where such conversations are conducted, and by whom. Consequently, the literature suggests it has not been systematically adopted in practice.^13^

To date, the majority of evidence for paediatric advance care planning is derived from adult populations,^11^ which does not recognise the substantial differences in terms of competence, legalities and degree of parental involvement. Existing research on paediatric advance care planning has focused on implementation, effectiveness^8,14–22^ and the development of programs and documentation. It can be argued however that there has been less attention given toward the process of initiation of advance care planning in practice. According to Van der Steen et al.,^23^ in their work with patients with dementia, initiation of an advance care plan refers to starting a discussion/decision making process, not necessarily resulting in concrete plans. Studies undertaken to date recognise that health care professionals are ideally placed to initiate such discussions, however they are often reluctant to do this due to difficulties in prognostication and fears that parents lack understanding or are not emotionally ready to engage.^8,15,18,24–27^ Although parents and minors are at liberty to start these discussions, the onus is on professionals to respond to parental and patient cues, or to ensure the conversation is started. Parental expections are that clinicians should take the lead.^28^ Whilst the time and manner in which advance care plan discussions are initiated is recognised as ‘the critical juncture, upon which all else hangs’,^29^ (p2) there is a paucity of data regarding the factors influencing the initiation of paediatric advance care planning from the health professional perspective.

### Aim

To appraise and synthesise current evidence regarding the factors influencing initiation of paediatric advance care planning discussions from the health professional perspective.

### Methods

#### Design

A systematic integrative review using guidelines developed by Whittemore and Knafl.^30^ This enabled the combination of diverse methodologies, providing a comprehensive review of the topic.^31,32^

### Search strategy

A search for existing literature to identify relevant papers on the initiation of advance care plans for children and young persons (<18 years) by health professionals was conducted using five online databases: CINAHL (EBSCO), MEDLINE (Ovid), EMBASE (Ovid), PsycINFO (Ovid) and Scopus.

Informed by previous work by Van der Steen et al.^23^ initiation was defined as (i) starting a discussion, not necessarily resulting in plans, (ii) starting the decision making or a decision-making process, i.e., actual planning of care or (iii) starting a written Advance care plan or Emergency care plan to be shared with health professionals, emergency services, educationalists etc. Multiple search terms were used informed by the literature.^8,14–21,33–37^

The search was extended from studies which were exclusively about initiation to include papers which included initiation as part of wider discussion. Papers on specific components of paediatric advance care planning such as end of life decision making and decisions on withdrawing and withholding treatment and resuscitation, which referred to initiation were also included. Keywords included: Advance care plan, Children, Palliative care, End of life care, Health care professionals, decision making, conversations, discussions or initiation. Full details can be found in (Appendix 1).

Medical Subject Headings (MeSH) and Boolean terms were used to efficiently identify the most relevant data, alongside free text, synonyms and truncation (Table 1) The search, screening and selection, was undertaken independently by two authors (KC & FH) and differences were mediated by a third reviewer (SM). The search was completed in October 2019 and was not limited by year of publication.

**Table 1.** Search terms - Integrative Literature Review.

**Key search concepts and terms and Boolean operators** – a combination of Medical Subject Headings (MeSH) and keywords were used. **Advance care planning** *and* **Child** *and* **Palliative care** *and* **Health care professionals** *and* at least one of the following **Decision making** *or* **Initiation.**			
Key search concepts	Example terms	Key search concepts	Example terms
Advanced Care planning	Advance care (plan or plans or planning), living will, advance (directive or decision).	Health professional	Nurse, paramedical personnel, physician, medical personnel
Child	Child, Adolescent, Infant	Decision making	Decision making, choice behaviour, share decision making
Palliative care	End of life care, Terminal Care, Hospice	Initiation	Initiat*, Conversation* or communication fishing questions, Trigger, Question prompt list, Prompt, Discus*, Talk, Converse, Debate, Confer, Deliberate, Consider

* Truncation symbol.

**Figure 1.**
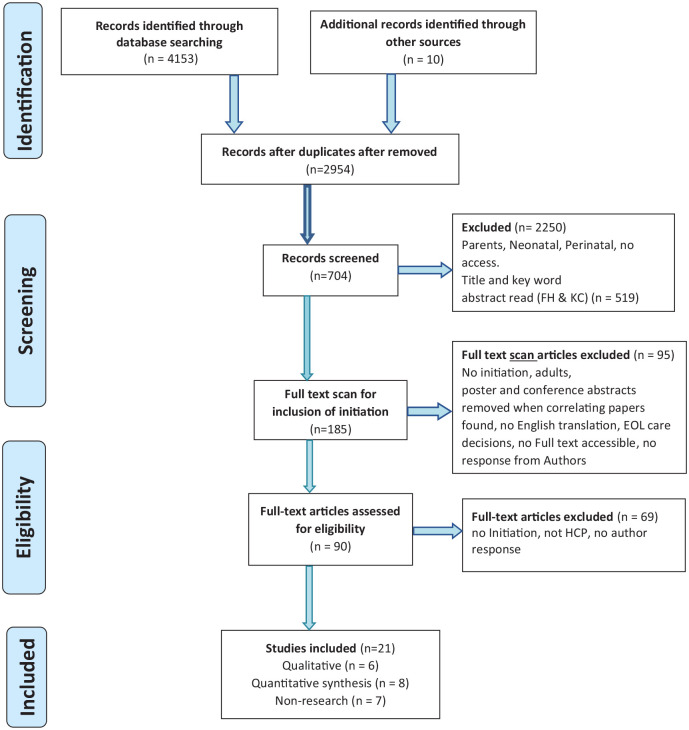
PRISMA Initiation of paediatric advance care planning integrative literature review. *PLoS Medicine* (OPEN ACCESS) Moher D, Liberati A, Tetzlaff J, Altman DG, The PRISMA Group (2009). Preferred Reporting Items for Systematic Reviews and Meta-Analyses: The PRISMA Statement. *PLoS Med* 6(7): e1000097. doi:10.1371/journal.pmed1000097.

A grey literature search of Ethos, Proquest, Open Grey, Prospero, Agency for Health Care Research and Quality, Google Scholar and Research Gate was also undertaken. Members of the International Children’s Palliative Care Network ICPCN (*n* = 1842) were contacted by e-mail to identify grey papers and guidance to ensure the search process was fully complete. Additionally, reference lists of relevant studies were hand searched. The search resulted in a sample of 4153 articles. Considering the inclusion and exclusion criteria, 21 studies remained (see Figure 1).

### Inclusion and exclusion

Following paper identification and de-duplication, titles and abstracts were screened, and full papers were assessed for eligibility.

The inclusion and exclusion criteria (Table 2) were applied to ensure only those papers applicable to the review aim were included. Neonatal and perinatal studies were excluded following a team discussion as deemed to be a highly specialised area requiring a separate search. Text and opinion papers were included if specific to the inclusion criteria.

**Table 2.** Eligibility criteria.

Inclusion criteria	Exclusion criteria
Papers specific to Initiation of advance care planning or any of the constructs linked to advance care planning such as ‘end of life care’, ‘withdrawal or withholding treatment’, ‘resuscitation decisions’, ‘wishes and hopes’.	Studies on parents
Empirical studies, (quantitative and qualitative and mixed method), theoretical literature, reviews, expert opinion and consensus reports where initiation specifically identified.	No full text version received from the corresponding author following two request emails
Full text studies published in any language with English translation available online	Neonatal and perinatal studies
Studies published up to 24th October 2019	
Studies in Children and young people <18 years old or those which separate data in this age group.	
Studies in Hospital, community or Hospice setting	

### Quality appraisal

Two reviewers (KC&FH) independently appraised the methodological quality of all the papers prior to their inclusion in the final review using Critical Appraisal tools from the Joanna Briggs Institute (JBI): for qualitative studies,^38^ for quantitative^39^ and for non-research text and opinion^40^ (Appendix 2). The standardised JBI tools use a comprehensive checklist with Yes, No, Unclear and Not Applicable as possible answers to 9 or 10 questions such as ‘Does the source of opinion have standing in the field of expertise?’ Findings are extracted and assigned a level of credibility.^41^ The methodological quality was assessed by assigning low (a score below 49%), medium (50–74%) or high (75+%) score. Scores were computed by counting the number of ‘Yes’ answers and expressing them as a percentage of questions in the tool (Appendix 2) to ensure fair comparison as the number of questions in the tools varied. Non-research (text and opinion) had five high and two medium, quantitative three high, four medium and one low and qualitative four high and two medium scores. No studies were excluded based on the ascribed quality rating although, it was included as a variable in the analysis stage and, in general, those of lower rigour contributed less.

### Data extraction and analysis

Data was extracted from the final papers independently by two reviewers (KC&FH) using a generic data extraction form and disagreements mediated by a third reviewer (SM) (Table 3). The data extraction process was based on the four stages identified by Whittemore and Knafl^30^ that is, data reduction, data display, data comparison and conclusion drawing. Finally, given the diversity of methodologies, the data were synthesised using constant comparison method^42^ which facilitates the identification of patterns, variations and relationships.^43^ This resulted in **four** final themes being identified: (1) Timing of initiation; (2) ***What makes an initiator, (3) Professionals perceptions and (4) Prerequisites to initiation.***

**Table 3.** Data extraction table.

Qualitative						
Author(s) country	Study aim (s)	Sample	Research design	Analysis	Key findings	Limitations
Henderson, et al.,^44^ Australia	To identify what paediatric healthcare professionals consider important when preparing for an EOL discussion.	Convenience sample *n* = 36 Medical, Nursing, AHP	Group interview	Descriptive content analysis	Themes identified: communication, healthcare professional perspectives, interdisciplinary team role, patient and family perspectives, practical issues, addressing mistakes, and healthcare professional education.	All participants had means to post anonymous comments but not all spoke at the interview. Results are from staff in one Australian state. Data saturation may not have been attained.
					Conflict makes it more difficult.	
					Acknowledging own anxiety and the uncertainty of each and every case.	
					Timing has to be right for the family rather than health professionals	
					Ask the parents: ‘‘are you ready to have this conversation about. . .’’	
					Ensure private environment.	
Hiscock and Barclay,^45^ United Kingdom	To investigate views and experiences of health professionals on discussions about advance care planning with teenagers and young adults with life-limiting neuromuscular diseases.	Health professional in adult and child health (6 different professions within neuro, respiratory, general and pall care).	Nine 1:1 Semi-structured interviews	Thematic content Analysis	Who: Those health professionals with long-term relationship with parent are best.	Small sample size with a wide range of health professionals
					Where: Home best.	
					When: Progression of disease was the main factor for initiation but this was problematic as resulted in delay and therefore less time for discussions.	
					Barriers: Parent/patient not ready/block discussion. Health professional themselves not ready. Organisational factors such as transition. Indicators for starting discussions such as cues and questions from patient/family or their answers to health professionals cited.	
					Although deterioration in NMD follows a predictable pattern there was no agreed consensus with health professional re advance planning.	
Jack et al.,^46^ United Kingdom	To explore health care professionals’ views and experiences of paediatric advance care planning in hospitals, community settings and hospices	Purposive sample *n* = 21. Dr, Nurses, AHP, Bereavement, C/A, Midwives	Naturalistic interpretative design. Semi-structured interviews.	Thematic Content Analysis	Themes identified:	Sample only included professionals who had been directly involved in the end-of-life care of children during the specified time frame.
					The timing of planning conversations, including waiting for the relationship with the family to form; the introduction of parallel planning; avoiding a crisis situation.	
					Supporting effective conversations around advance care planning, including where to have the conversation; introducing the conversation; and how to approach the topic encompassing the value of advance care planning and documentation for families.	
					How to introduce the conversation was an important consideration for the participants. Example was given of how they approach families to initiate paediatric advance care planning conversations when a child is showing signs of deterioration and another emphasised the emotional value of the advance care planning process for the family. Picking up family readiness cues was noted.as part of timing the initiation of paediatric advance care planning.	
Lotz et al.,^24^ Germany	To investigate the attitudes and needs of health care professionals with regard to paediatric advance care planning	Purposeful sampling *n* = 17 Doctors, Nurses, Social health professional	1:1 Semi-structured interviews	Qualitative content analysis MAXQDA-10 software	Perceived as helpful by providing a sense of security and control, improving quality of care and ensuring respect of patients’ and parents’ wishes.	The convenience sample of health professionals which was known to the researcher may have biased the results. The area in which may have resulted in an overly advanced view of the current paediatric advance care planning practice and health professionals’ attitudes toward paediatric advance care planning which would make the study difficult to replicate in less advanced areas.
					Problems identified related to professionals’ discomfort and uncertainty regarding end-of-life decisions and advance directives.	
					Timing – Identified early initiation of paediatric advance care planning shortly after diagnosing an incurable condition, but this was recognised as unrealistic in many cases and that family’s readiness was important.	
					Specific times to initiate – such as discharge at home or a severe deterioration of the child’s condition were indicated.	
					Paediatric advance care planning was noted to be an individualised process with continuity of staff qualified to facilitate, the need for multi-professional meetings and for professional education all requirements.	
					Difficulties identified: health professional discomfort with paediatric advance care planning, unclear responsibilities, uncertain prognoses, difficulties in initiation, problems identifying the child’s wishes, the burden for parents, paediatric advance care planning document limitation, uncoordinated communication and insufficient implementation within health care system.	
Mitchel and Dale,^15^ United Kingdom	To explore the experiences of senior medical and nursing staff regarding the challenges associated with Advance Care Planning in relation to children and young people with life-limiting illnesses in the Paediatric Intensive Care Unit environment and opportunities for improvement.	Purposeful sampling *n* = 14 Consultants and senior nursing staff.	1:1 Semi-structured interviews	Thematic Content Analysis	Themes identified: Recognition of an illness as ‘life-limiting’; paediatric advance care planning as a multi-disciplinary, structured process; the value and adverse consequences of inadequate paediatric advance care planning and additional difficulties of advance care plan at transition points.	Conducted within one PICU in England and included a relatively small number of participants.
					Benefits: Opportunity to make decisions regarding end-of-life care in a timely fashion and in partnership with patients, where possible, and their families.	
					Barriers: Recognition of the life-limiting nature of an illness, illness trajectory and gaining consensus of medical opinion as key barriers to initiating paediatric advance care planning. The multidisciplinary, dynamic nature of the process, time constraints, conflicting clinical demands and lack of formal training in communication skills were also barriers and specific to the PICU setting, a lack of established rapport with the family was identified as a problem.	
Zaal-Schuller et al.,^47^ Netherlands	To investigate the experiences of the parents and the involved physician during the end-of-life decision-making (EoLDM) process for children with profound intellectual and multiple disabilities (PIMD).	Various recruitment strategies *n* = 14 Doctors and parents of children with PIMD.	1:1 Semi-structured interviews	Qualitative data analysis software, MaxQDA	Themes identified: The influence of previous healthcare encounters, Anticipation and timing of the EoLDM process, Provision of information and advice, Reasons for disagreement, Contributions to decision-making, The final decision maker.	Fathers’ perspective is lacking. Recall bias is possible.Parents could have a more positive view about the EoLDM process if their child was still alive.In cases of disagreement doctors responded broadly making the comparison between the experiences of parents and physicians more difficult.Generalizability limited as only Dutch hospitals studied.
					Facilitators: Relationship with the family which Physicians put a lot of effort into to achieve and maintain. Parents knowledge of the medical conditions and their experiences with treatments during previous critical illnesses which was recognised by doctors as Parents of children with PIMD being experts and allowed more influence in decision-making.	
					Barrier: Previous negative healthcare experience. Many physicians had an idea about how parents felt about EoLD, they found it very difficult to identify when parents were ‘ready’ to discuss these decisions. Uncertain prognosis and unforeseen complications. Parents’ difficulties fully understanding information. Parents wishing for ‘everything to be	
					Done, even treatments considered futile and the opposite.	
					Both parents and physicians preferred a shared decision-making approach though there were differences in the understanding of what SDM was.	
					Disagreements were not uncommon but strengthened the decision-making process as they were discussed.	
					Timing: Acute deterioration	
					Reviewed if improvement/deterioration and at annual reviews	
Quantitative						
Author(s) country	Study aim (s)	Sample	Method	Analysis	Results	Limitations
de Vos et al.,^48^ Netherlands	To investigate how Dutch paediatric specialists, reach end-of-life decisions, how they involve parents, and how they address conflicts.	*N* = 138 Medical specialists’ paediatric intensivists, oncologists, neurologists, neurosurgeons, and metabolic paediatricians	National cross-sectional survey.45 Questions.	SPSS – Significance level of .05 used.	End-of-life decision discussed with colleagues before discussing it with parents. Initiate discussion re LST pre-crisis situations. 25% use local guidelines. Initiated by the medical team in 75% of the cases in 4% by the parents, and in 21% by both.Decision making Paternalistic half – parents informed and asked, ¼ Parents informed but not asked for their permission. 1/4 advised parents and they decided. The chosen approach is highly influenced by type of decision and type and duration of treatment. Conflicts within medical teams arose as a result of uncertainties about prognosis and treatment options. Most conflicts with parents arose because parents had a more positive view of the prognosis or had religious objections to treatment discontinuation. All conflicts were eventually resolved by a combination of strategies.	Number was low for a national survey. Results are the opinions of respondents, not on direct observations. Only Doctors’ perspective.
Durall,^27^ USA	To identify barriers to conducting advance planning discussions for children with life-threatening conditions	E-mail invitations *n* = 266 Doctors and Nurses ICU areas and oncology in two Children’s hospitals.	Electronic Survey – 148 questions derived from clinician and parental focus groups. De novo and existing questions. Pilot tested.	SPSS. Pearson χ^2^, Mann–Whitney *U* tests.	Response rate 54%.	Limited generalisability as only Doctors and nurses from three departments within one hospital. Participation may have been influenced by experience. Patient and parental perspectives not studied.
					Timing: 71% of clinicians believed that ACD typically happen too late.	
					92% believed that a discussion regarding overall goals of care should be initiated upon diagnosis or during a period of stability. 60% reported that these discussions typically take place during an acute illness or when death imminent.	
					Who: Only 1% of clinicians believe that patients or their parents should initiate ACD; the majority felt that responsibility rests with one of the patient’s physicians or advance practice nurses.	
					Barriers: Unrealistic parent expectations, differences between clinician and patient/parent understanding of prognosis, and lack of parent readiness to have the discussion. Nurses identified lack of importance to clinicians and ethical considerations as impediments more often than physicians. Physicians believed that not knowing the right thing to say was more often a barrier. There are also perceived differences among specialties. Cardiac ICU providers were more likely to report unrealistic clinician expectations differences between clinician and patient/parent understanding of prognosis as common barriers to conducting ACD.	
Forbes,^49^ Australia	To better understand current attitudes and practices relating to discussions concerning the withholding and withdrawing of life-sustaining medical treatment (WWLSMT) among medical staff in the paediatric setting.	N+ 385 Doctors and Medical Students.	Anonymous online survey	SAS	Response rate 42%.	One Hospital in one state. Response rate was only 42.1% of which 50% were junior Doctors.
					Majority of Junior Doctors are uncomfortable discussing WWLST.	
					Experience led to more comfort in WWLST discussions with clinical acumen, communication skills and the observation of more senior colleagues also rated highly. Confidence in having WWLST discussions correlates with experience.	
					Most learned through experience and by observing more senior colleagues, with 58% of Junior and 35.8% of Senior staff having no specific communication training regarding WWLSMT.	
					Barriers: concerns about family readiness for the discussion, prognostic uncertainty, family disagreement with the treating team regarding the child’s prognosis/diagnosis and concerns about how to manage family requests for treatments that are not perceived to be in the child’s best interests.	
Harrison et al.,^50^ USA	To understand communication among health care professionals regarding death and dying in children.	*N* = 133 Nurses, Doctors Psychosocial Clinicians. One USA Hospital.	Survey – paper. Doctors – 24 items, nursing – 27 items, psychosocial – 56 items.	Spearman’s correlation, a Multivariate Analysis of Variance (MANOVA) and Analysis of Variance (ANOVA)	Response rate 90%. Comfort in discussions: Health care professionals who felt comfortable discussing options for	There may have been Selection bias as participants had the option to participate – those more likely to be interested in this topic would participate. Potential recall bias. Including the parents’ perspective absent. Definition of previous training could have been clearer.
					end of life care with colleagues also felt more comfortable: initiating a discussion regarding a child’s impending death with his/her family discussing options for terminal care with a family, discussing death with families from a variety of ethnic/cultural backgrounds,guiding parents in developmentally age-appropriate discussions of death with their children, identifying and seeking advice from a professional role model regarding management concerns, or interacting with a family following the death of a child.	
					Who: Doctors were more likely and were more comfortable than other staff to initiate discussions.	
					Training: Health care professionals that received formal grief and bereavement training were more comfortable discussing death.	
Kruse et al.,^51^ USA	To evaluate the extent to which paediatric providers have knowledge of code status options and explore the association of provider role with (1) knowledge of code status options, (2) perception of timing of code status discussions, (3) perception of family receptivity to code status discussions, and (4) comfort carrying out code status discussions.	*N* = 263 nurses, trainees, and Doctors	Cross-sectional survey. Hard copy. Instrument contained 10 items	SAS	Response rate 90%. Knowledge of code status (resuscitation) options was consistently low – which differed to perceived knowledge of which Doctors perceiving themselves as having the greatest knowledge.	One site, which may limit the generalisability of findings.Provider’s perspective only; no study of patient/parent perspectives. Study did not look at differences in specialities and differences in stage of experience/training not accounted for.
					Comfort. 58.2% of Doctors have the highest comfort level when discussing code status. Nurses and trainees were similar.	
					Family receptivity to discussions – Doctors and trainees perceive families to be more receptive to discussions than nurses do.	
					**Timing:** Nurses perceive Timing of discussions to be too late (63.4%) and most Doctors (55.6% feel they are timed right with none thinking they are too late.	
Sanderson et al.,^25^ USA	To identify clinician attitudes regarding the meaning, implication, and timing of the DNR order for paediatric patients.	E-mail invitations *n* = 266 Doctors and Nurses ICU areas and oncology in two Children’s hospitals.	Electronic Survey – 148 questions derived from clinician and parental focus groups. De novo and existing questions. Pilot tested.	SPSS. Pearson χ^2^, Mann–Whitney *U* tests.	Response rate 54%.	Study involved clinicians from only three departments within one hospital, therefore, may have limited generalisability. No patient and parental perspectives.
					**Timing:** There is a defined difference between what health professional believe is the correct time to initiate DNR discussions, *n* = 99 at presentation and *n* = 79 when stable as opposed to what is happening in practice acute illness *n* = 80 or when death imminent *n* = 131. Most clinicians reported that resuscitation status discussions take place later in the illness course than is ideal.	
					**Barriers:** unrealistic parent expectations (39.1%), lack of parent readiness to have the discussion (38.8%), and differences between clinician and patient/parent understanding of the prognosis (30.4%) were identified as most common.	
					There was substantial variability in the interpretation of the DNR order. Most clinicians (66.9%) believe that a DNR order indicates limitation of resuscitative measures only on cardiopulmonary arrest. In reality, more than 85% believe that care changes beyond response to cardiopulmonary arrest, varying from increased attention to comfort to less clinician attentiveness.	
Basu and Swil,^11^ Australia	To assess physicians’ experiences and education regarding paediatric advance care planning. To assess barriers to advance care plan initiation, including the adequacy of exposure and education regarding advance care planning and whether practitioners would deem improved education and resource provision useful.	*N* = 93 Paediatricians, intensivists and advanced trainees	Electronic Survey	Microsoft Excel	Patients with life-limiting conditions are encountered frequently, with 57% of respondents caring for at least 10 such patients during the last 2 years.	Small sample size and a single hospital site may reduce generalisability.
					**Who:** 46% felt that multidisciplinary teams were the most appropriate to initiate advance care plan discussions	
					**Barriers:** Prognostic uncertainty and lack of experience and education were identified as barriers by 43% and 32%, respectively. Personal clinician factors and relationships with families.	
					**Training:** Exposure to ADVANCE CARE PLAN and education during training inadequate	
					**Time:** 64% of respondents felt that ADVANCE CARE PLAN discussions should occur early around the time of diagnosis or during a period of stability; however, 57% observed discussions occurring late in illness after multiple acute, severe deteriorations.	
Bradford,^52^ Australia	To define optimal components of an early paediatric palliative care consultation.	*n* = 19 Medical physician, Nursing, Allied health	Delphi study	Percentage frequencies and Standard deviation	Response rate 19.	Response rate – low for survey but appropriate in a Delphi study. No accepted benchmark for consensus. Only experts from Australia and New Zealand.
					Priorities: establish rapport with the family, establishing the family’s understanding of palliative care; symptom management; an emergency plan; discussion of choices for location of care, and a management plan. Components considered suitable to defer to later consultations, or appropriate to address if initiated by family members, included: spiritual or religious issues; discussion around resuscitation and life-sustaining therapies; end-of-life care; and the dying process.	
Non-research						
Author(s) country	Author details	Content		Main information relating to review – Limitations are that it is the authors view although based on experience and often research papers.		
Harrop,^53^ United Kingdom	Health professionals from one UK Children’s Hospice and two bereaved mothers who used advance care planning provide their views. Research to back up practice and experience.	Professional and user information – Authors share experiences, in the context of national guidance on the use of advance care plans.		Advance care planning influences the treatment received and improved their experience of care. Recognised as a difficult area of practice for healthcare professionals. Health professional and families appear to benefit when the process is fully informed, and the child and family are actively involved. Honesty about area of clinical uncertainty and an understanding of the dilemmas faced both by clinicians and families are most likely to lead to a successful outcome both for the advance care plan and ultimately for the care agreed within it.		
				**Time** – When it best suits the family, but it depends on diagnosis. Sometimes it is clear – e.g. change in goals. increasing intercurrent illnesses.		
				**How** – Warning shot, time to think, additional resources e.g. leaflet, blank advance care plan documentation.		
Haynes et al.,^54^ United Kingdom	Dr’s and Nurse in Neurodisability and PPC	Step by step guidance on introducing and creating paediatric advance care planning’s for child with severe disabillity		Increase number of LL/LT children dying in pICU. The importance that the family should have a paediatric advance care planning document with emergency care plan which has (i) Emergency plans, (ii) wishes for EOL and non-medical choices. Who: A trusted health professional well known to child and family. Prerequisites: Early identification of the life limiting condition, open discussion regarding prognosis, acknowledgement of uncertainty, parallel planning, early introduction to PPC services, paediatric advance care planning preferences. Preparation by health professional: Clear knowledge of condition and treatment. Discussion with other health professional involved. Introduction of paediatric advance care planning ‘idea’ to family. Suggested phrases. Options available and discussions on these. Information on EOL, death and following death preferences. How the document should be agreed, updated and shared and how to approach non-agreement.		
Mack and Joffe.,^55^ USA	Two medical doctors based in Paediatric Haematology and Oncology.	Considers communication about prognosis in the context of the patient–clinician relationship which in paediatrics is unique due to the tripartite relationship of parent, child, health professional.		**Health professional perceptions** – That prognostic information will cause patients emotional distress, take away hope.		
				Could be inaccurate, may cause the patient to ‘give up’ and that some patients do not want to know what is ahead. Some believe that those from minority racial or ethnic backgrounds may be less likely to want prognostic information.		
				**How** – honest and supportive conversation. Doctors who face considerable prognostic uncertainty can begin conversations by using language that is open to multiple possible outcomes long before acute deteriorations necessitate urgent decision-making.		
				**Time** – Doctors often do not know when to initiate paediatric advance care planning discussions particularly when dealing with uncertain prognosis. Life limiting illnesses are diverse and often have long, waxing and waning courses, paediatricians’ opinions about the optimal timing of referrals to palliative care vary widely, potentially fostering divergent practices in discussing prognosis. Inexperienced with communication about end-of-life care, which may lead to delays in conversations about prognosis and care preferences.		
Pao and Mahoney,^56^ USA	Psycho-oncology Doctor and Research Assistant.	Paper comments on the preparation, rationale, and benefits of paediatric advance care planning discussions in a developmentally sensitive manner with adolescents with LL/LT conditions.		Health professionals must look at their own readiness to engage by taking a self-inventory, learning communication skills, and understanding individual barriers.		
				Talking with adolescents who have a life-threatening or life-limiting illness is one of the most difficult tasks a health care provider (health professional) can undertake.		
				Adolescents want to be included in medical decision-making through the illness trajectory including making decisions around end-of-life.		
				Prognosis is not necessary before initiating advance care planning discussions.		
				**Time:** not easy to know when is best to initiate advance care plan conversations with patients and families. Balancing act between the adolescent’s readiness and that of their family’s and, separately, the health professional’s readiness.		
				Suggested timing questions for health professional.		
				Readiness assessment probing by asking adolescents whether end-of-life conversations would be helpful or upsetting, and if they feel comfortable discussing preferences when treatment options become limited.		
Sidgwick et al.,^57^ United Kingdom	Paediatric Intensive care (pICU) & Paediatric palliative care (PPC) Doctors	Parallel planning in paediatric critical care		Repeated admissions to pICU of LL children with often death whilst receiving critical care. Acknowledges difficulty in initiation. Who – Identifying right competent trusted professional a challenge. Learn by observing experienced colleagues. Advocates more children with LLCs should be offered parallel planning before pICU admission but acknowledges that often parents not ready to make decisions or change their mind in a crisis.		
Tsai et al.,^58^ Canada	Physicians and Ethicists	Position statement on advance care planning in children. To assist health care practitioners to discuss advance care planning for paediatric patients in varied settings.		Health professionals should educate themselves to be comfortable initiating discussion. Advance care planning is part of the standard of care. Wishes regarding emergency and life-sustaining therapies should be documented		
				**Who:** Health professional responsibility to initiate these discussions.		
				**When:** Should occur early and regularly before crises arise, and as the goals of care are clarified or change over time.		
Wiener et al.,^59^ USA	Psycho-oncology Doctor and Research	Commentary on progress in the area. Focuses on how healthcare professionals can approach advance care planning (advance care plan) with adolescents and young adults (AYA), involve their family members, and engage the entire health care team.		**How:** Assess advance care plan discussion readiness. Tailor to the individual needs of the AYA and family.		
				**Time:** AYA and family must acknowledge that cure may not be possible. When in relatively stable health and willing to engage in conversations about future treatment and lack of future treatment.		
				**Who:** Member of the healthcare team who has the confidence and trust of the AYA and their family and who understands the specific psychosocial needs. Doctors, by nature of their role, are uniquely responsible for relaying bad news.		
				**Preinitiation:** family must acknowledge that cure may not be possible. They also need to be amenable to explore the AYA’s thoughts, preferences, and/or goals.		
				**Barriers:** providers feeling unprepared or without adequate skills to guide EOL discussions,		
				**Health professional perceptions:** parental concern that discussing plans, including life support options or presenting an EOL planning document may send the message that the medical team wishes to withdraw care or that death is imminent.		

## Results

The Preferred Reporting Items for Systematic Reviews and Meta-Analyses (PRISMA) 2015 guidelines for article selection^60^ was used to report this review.

In total 21 papers were included in the final analysis. More than half of the papers were about the generalities of paediatric advance care planning ^11,15,24,27,45,46,53–56,58,59^ with only one study focused exclusively on initiation of advance care planning in children.^11^ The reminder of the papers centred on discussions and decision making about end of life,^44,47,48,50,57^ resuscitation,^25,51^ withdrawal of treatment^49^ and one on components of early paediatric palliative care consultations.^52^ All the papers stemmed from developed countries, seven from the United States,^25,27,50,51,55,56,59^ four from Australia^11,44,49,52^ one each from Canada^58^ and Germany,^24^ two from Netherlands^47,48^ and six studies from the United Kingdom.^15,45,46,53,54,57^

Fourteen papers were empirical i.e.: quantitative (*n* = 8)^11,25,27,48–52^ of which one was a consensus-based method,^52^ qualitative (*n* = 6).^15,24,44–47^ Seven papers were professional reviews.^53–57,59^ or position statements.^58^ The earliest published paper was 2008 with nine published since 2017. The most common settings for initiation were intensive care^15,25,48,51,57^ and oncology wards^25,27,48,51^. Information re country, year, setting and sample for the included papers are available in Appendix 3.

Four key themes emerged which were found to influence the initiation process, *(1) Timing of initiation, (2) What makes an initiator, (3) Professionals perceptions and (4) Prerequisites to initiation*.

### Theme 1: The timing of initiation

All papers in the review advocated that paediatric advance care planning be undertaken, however discrepancies in the initiation process were evident with regards to timing. There is differing evidence on the appropriate timing and diverse triggers used for the initiation and/or delay of starting advance care planning conversations. Timing in all the papers referred to the stage in the illness trajectory, with only one referring to the time of their hospital experience i.e. discharge.^24^ None of the papers indicated time of day for either family or professionals being significant and only two indicated that the professional needs to ensure enough time available^15,57^ with the time required acknowledged as a challenge^59^ along with the acknowledgement of other clinical demands on the professionals.^15^ Critically, one paper states that advance planning discussions in children need not necessarily be lengthy^56^ if the groundwork of the relationship and permissions has been established indicating the importance of initiation.

In total, 20 papers^11,15,24,25,27,44–49,51–59^ reported on the stage of the illness trajectory for the initiation of advance care plan discussions. A focus on the correct ‘time’ and the ‘right time’ for both health care professionals and the patient/family, underpinned this debate. Most studies advocate discussion to be started early, ideally close to diagnosis.^11,24,25,27,46,54,58,59^ However the stated timing triggers for initiation varied from occurring when the child was stable^11,25,27,57–59^ or to when the goals of care changed,^53,58^ to responding to physical deterioration and not being expected to survive the next 12 months.^54^ No paper however, provided clarity on what ‘early’, ‘close to diagnosis’, ‘end of life’, ‘late’ and ‘following deterioration’ means in practice though these were terms frequently used.

‘Early’ initiation was viewed as beneficial for the health professional, family and child. For example, it was believed to enable parallel planning to occur,^46,57^ relationships to be developed between the health professional and family^11,15,44,46,47,52,53,56,59^ and potentially result in less aggressive intervention and an increase in palliative care support.^55^ Several studies indicated that starting discussions in a proactive manner enabled a staged approach with the more ‘difficult’ components of advance care planning being discussed when health professionals and families have had time to get to know each other and develop a relationship.^11,15,44,46,47,52–54,57,59^ Using ‘natural’ triggers such as following an episode of deterioration,^47,55^ prior to paediatric intensive care admission^15,55^ and families’ asking leading questions^46^ was indicated as an opportunity to introduce the topic or to assess family readiness to have an advance care planning discussion.

In practice however, it was recognised that paediatric advance care planning often occurs late, often when death is close,^11,25,27,45,51^ triggered by a crisis and often after multiple deteriorations.^11,25,27,47^ Several factors were cited as reasons to avoid starting these conversations, such as uncertain prognosis^11,15,24,27,47,49,55,56,58^ or lack of health care team consensus prior to speaking to parents.^15,44,48,57^ However, Henderson^44^ warned awaiting consensus may result in a further delay in the initiation of advance care planning discussions. In addition, perceptions that families are reluctant to discuss future care decisions prior to physical deterioration^24,25,27,45–47,49,54,55,57^ and family dynamics^25,27,56,58^ resulted in delays in conversations occurring. The presence of disagreement, or fear of creating conflict between the health care team and family^24,44,47–49,56^ and within the family^25,27,56,58^ were also identified as influencing factors.

One paper recognised the need for families to process news such as diagnosis before being ready for advance care planning^24^ and others identification of specified situations where extra time would be needed – that of differences in language and religion^48^ whilst others cautioned on ensuring enough time was made available.^15,48^

### Theme 2: What makes an initiator?

#### Lead taker

Whilst the literature recognises that parents play an essential role in the advance care planning process,^47^ this role is less clear when it comes to initiation.^27,53,58^ At the initiation stage the role of health professionals was viewed as vital, with the onus on them to start the discussion or at least inform parents accurately about paediatric advance care planning.^11,15,27,44,45,48,53,54,57–59^ However, no consensus on which professional group was best placed to do this was reported, instead it included doctors,^27,54,58,59^ advanced nurse practitioners,^27^ or members of the multidisciplinary team (MDT) without specifying which member^11,15,44,53^ and the difficulty of identifying the ideal health professional in the team acknowledged.^57^

Rather than naming a specific health professional who has responsibility, several papers^11,53,56–58^ present criteria for appropriateness of the professional e.g. that it should be based upon quality of health professional and family relationship.^53,56–58^ However, others suggest that the health professional should be the primary professional who has had responsibility for majority of care,^11,58^ or who is an expert in the disease, its pathway and the impact on the child’s quality of life.^53^ Only one study^57^ identified requirements for an ideal initiator – motivation, time, emotional capacity, expertise in the child’s condition and palliative care knowledge. Doctors were identified as the health professional who most often undertake these conversations^49,50^ however the nurse (grade unspecified) would provide the confirmation to the doctor of the patient’s physical decline and family dynamics which then acted as a catalyst for action.^15,47^

The choice of the doctor to start such conversations was justified based on the evidence that although they felt discomfort addressing paediatric advance care planning^24,44,51,57,59^ they were often more comfortable initiating discussions rather than nurses or psychosocial staff.^50,51^ However, reticence on the part of the health professional, including doctors, to initiate conversations was evident.^24,44,51,59^

### Professionals’ learning processes

It was perceived a correlation existed between increased clinical exposure,^11,49,57^ knowledge and training,^50^ regarding the attitude and ease of approach of the health professional. Doctors were often hesitant to take the lead citing a lack of knowledge and training as key reasons to avoid taking the role.^11,15,24,44,51,59^ Reports suggest that health professional knowledge and practice were learned in an ad-hoc manner on the job from observations and discussions with experienced colleagues.^49,50,57^ Furthermore, formulating the message and knowing how to verbalise difficult conversations, specifically, knowing the right words to use was indicated as problematic in several studies.^11,24,27,44,46,53,55,56,58,59^ Three papers indicated that health professionals did not know the right words to use.^11,24,27^ Prompts and conversation starter examples were suggested in eight papers.^44,46,53–56,58,59^

### Approach strategies

Preparing not just themselves but the family member prior to starting an advance care planning conversation was suggested in one paper^53^ which advocated giving a warning shot that planning discussions would happen in the future. Another method of introduction was the use of parallel planning, identified in two papers^46,57^ where palliative care is introduced alongside curative care and the advance care plan reflected various potential directions the illness/treatment may take the child. Another introduction suggestion was extoling the benefits to parents, such as not having to repeat the same story every admission or to new health professionals.^46^

### Theme 3: Professionals perceptions

Central to the initiation of advance care planning discussions were health professionals’ sensitivities of family reactions and receptiveness, and their own perceptions on palliative care.

### Professionals’ perceptions of families

Whilst the inclusion of parents in open and honest discussions was advocated,^53,55,59^ health professional’s perceptions of the family reaction^55^ and concerns about causing distress,^11,46,49,55^ taking away hope^24,55,56,58,59^ or broaching topics for which the family are perceived either not to be ready^24,25,27,46,47,49^ or do not wish to engage in,^55^ impacted on the initiation of conversations. Moreover, health professional’s assumptions of a family’s lack of understanding of the diagnosis, prognosis and treatment^24,25,27,44,47,52,55^ meant that this also acted as a deterrent to start discussions. Health professional worries about offending families of other religious and cultural backgrounds to their own^11,15,24,49,55^ was also found to impact who takes the lead, timing, and the message delivered. The cultural, religious and belief systems of the health professional was recognised as influencing the process, with research suggesting their attitude to death and advance care planning could influence their ability, confidence and process of initiation.^11,55,56^ However, one Australian study refutes these claims suggesting health professionals lack of understanding of families’ religious beliefs was of greater concern.^49^

### Professionals’ perceptions of palliative care

Health professionals were also found to hold beliefs relating to exclusivity of palliative care treatment and active treatment^15,52,58^ and a lack of certainty of when to refer to palliative care.^55^ Many health professionals essentially viewed paediatric advance care planning as a decision-making process^24,47,48,53,55,56,59^ focusing on decisions relating to the withdrawal or withholding of life sustaining treatment and resuscitation.

### Theme 4: Prerequisites to initiation

Findings illustrate that numerous prerequisites play a fundamental role in initiation of advance care planning discussions.

Separate from the requirements identified regarding time of initiation and the need for consensus of professionals involved, other health professionals’ prerequisites to initiation were identified within the 21 papers including: Training,^11,15,24,44,51,59^ with formal training linked to increased professional’s comfort in discussing death with families;^50^ Associated with training, but not dependent on it, the possession of good communication skills^15,44,49,50,56,58^ was identified as a requirement; The need for parents to have an understanding of paediatric advance care planning prior to starting^54^ and to indicate their readiness to participate^24,25,27,46,53,54,56,59^ was identified as necessary. Three papers also indicated that, where appropriate, the patient must also indicate participation readiness;^45,56,59^ The need to have a clear diagnosis and prognosis or, in the absence of these, evidence of a deteriorating condition or imminent death.^25,27,47,49,54,59^

Communication about paediatric advance care planning was seen to be interdependent on other difficult conversations such as the need for open discussions of disease progression and prognosis, including prognostic uncertainties;^11,27,49^ Four papers indicated the need and importance for an appropriate physical setting for the initiation of discussions^24,44,46,56^ and specified the disadvantage of engaging in such conversations in a busy clinical environment, recommending the importance of planning the environment^44,46,56^ and that health professionals initiate the discussion away from the child.^44,46^

## Discussion

### Main findings/results of the study

This integrative review approach uncovered a scarcity of evidence on the initiation of paediatric advance care planning with only one study,^11^ focusing on this. There is diversity in practice across countries resulting in no international evidence base. There was no consistent practice regarding initiation, rather findings suggest this is a complex process influenced not only by actual issues such as diagnosis, or parent indication of readiness but also by perceived issues such as families potential negative reactions or that it was another professionals responsibility. The influence, if any, on the initiation process of the complexities of dealing with a varied range of diagnosis, family situations, parental obligation to protect and societal predisposition in favour of life^59^ that envelops paediatric clinical care requires further exploration.

Papers revealed three overarching and largely interrelated areas which in turn result in indecision.

In **timing of initiation** uncertainty of prognosis was an important factor that influences both the initiation and focus of advance care planning discussions. Similar to previous work^8,18,26,61^ prognostic uncertainty influenced the timing and acted as a key barrier to starting advance planning discussions. Whilst advance care planning discussions were advocated early in the illness trajectory,^11,24,25,27,46,54,58,59^ evidence suggests they were initiated in direct response to the physical deterioration of the child, which acted as a key trigger and catalyst. This may help to explain why some studies^25,48,49,51^ reported on the initiation of advance care plan discussions based on the medical/technical aspects of care and not the holistic values approach advocated in the literature and policy.^3,5,6^ Moreover as no paper provided clarity on timing it is important to identify if there ever is ‘a right time’.

### The making of an initiator

In the absence of a nominated leader for initiating advance care planning, uncertain qualities, skills and leadership influenced who took on the initiation role and how it was performed. For example, resistance to initiation was closely linked to health care professional’s own uncertainty in responding in a vulnerable situation.^10,44^ In the absence of tools and guidelines to assist professionals they relied on their instinctive feelings and perceptions to gauge a parent’s openness to engage in end of life discussions. This was further compounded by their lack of competence, knowledge and confidence about how to initiate, respond to and deal with such conversations.^11,15,24,44,51,59^

With regards to who takes the lead to initiate advance care plan discussions, some studies rationalised this as the doctor’s domain^27,54,58,59^ whilst others the responsibility of a specialist nurse.^27^ Yet there was no consensus on whose role or responsibility it was to lead such discussions resulting in the ‘bystander effect’ occurring in practice, where health care professionals from one discipline waited for other professionals, or indeed families, to start the conversation.^62^ Being part of a large group implied that no single person was necessarily identified as responsible for initiation therefore individuals could not be held responsible for inaction.^63^ The hope is that someone who knows the child, family, condition, symptoms more, who is better placed timewise and who has the experience and confidence, that is – the professional with the capability, opportunity and motivation will step up and lead the advance planning.

The importance of **professionals knowing themselves and knowing the families** was evident with the unpredictable nature of family reactions having an impact on both the timing and initiation approach reflecting previous work.^18,61,64,65^ Advance care planning discussions were viewed to be emotionally complex and fear of initiating conversations was expressed specifically and centred on the perceived negative parent reaction^24,46,49,55,56,58,59^ and concerns that parents would believe health care professionals were giving up on life extending treatments.^59^ Discussions which focused on emotional/quality of life issues where perceived as taking longer and therefore within the realities of practice they were avoided.^15,66^ Beliefs that parents wanted to continue to pursue disease directed therapies and that honesty about prognosis would contribute to undue distress,^11,46,49,55^ remove hope^24,55,56,58,59^ and/or offend the parent,^11,15,24,49,55^ led to an unwillingness to initiate such discussions. Yet this is in contrast to research involving parents which suggest that they want to be involved and indeed would prefer advance planning initiated earlier.^10,66–68^ A 25-year-old study^69^ indicated that all parents of life limited children, in particular parents who believed that professionals didn’t understand their needs, (parents) or the Childs, were especially keen on having written advance care plans. There was no evidence as to how professionals came to these conclusions or tested them such as checking readiness to participate or using tools such as ‘the care planning readiness assessment’.^70^

To counteract and respond to the uncertainty of who, how and when to initiate healthcare professionals developed pre-requisites to be in place, to facilitate the initiation. For example, prognosis uncertainty required an expert in the condition to be the lead role.^53,57^ The unpredictable outcomes of the parent and the professional’s lack of confidence could be tempered by having a relationship with the parents.^15,52^ However, regardless of the number of pre-requisites that exist, what is apparent is that initiating discussions about advance care planning is challenging and raises many dilemmas for healthcare professionals. In practice, professionals may need to realise that uncertainty may be unavoidable and inherent, and no universal guideline can address the unique situational, contextual, organisational and personal issues that surround such discussions. Harnessing, acknowledging and working with this uncertainty, through honest negotiations with parents, was recognised as necessary^53–55^ with the overall aim that such discussions are initiated, rather than delayed.

### Strengths and limitations

This is the first integrative review exploring the initiation of paediatric advance care planning from the health professional viewpoint. The methodology adhered to the PRISMA statement and the quality of all studies were critically assessed using methodological criteria. Although based on a comprehensive search and despite no geographic restrictions being placed on the search strategy all studies stem from developed countries with papers exclusively from only three continents (North America, Australia and Europe) which limits the generalisability of the findings. This review did not include the factors associated with parent initiation of advance care plans. Whilst comprehensive terms linked to initiation of paediatric advance care planning were used to guide the search it is recognised it may not have been able to capture all the available evidence. Recognition of the international heterogeneity in how paediatric advance care planning is defined, and analysed, questions the generalisability of the process and findings. This review was limited in that it included the initiation of discussions of components of paediatric advance care plans, such as treatment limitation, as well as papers specific to paediatric advance care planning. This broadening of the search was necessary due to the limited literature available specifically on the initiation of paediatric advance care planning and to recognise that many professionals see components, such as treatment limitations, as the focus of advance care planning rather than family and child goals and wishes with treatment decisions a component, not the main focus and entirety. Finally, this review only included papers with easily available translation into English therefore papers existing in other languages, were not included in this review.

### What this study adds?

This study reinforces previous studies on components of paediatric advance care planning and highlights the lack of evidence in the general topic and specifically initiation. An array of personal, social, cultural and organisational factors influences how, who and when paediatric advance care planning is initiated. Developing a rapport, professional knowledge of paediatric advance care planning, educating the parent and approval to talk on the topic are some of the factors outlined as important to consider when initiating paediatric advance care planning conversation with parents.

### Implications for practice, theory or policy

It is not possible to recommend effective ways of initiating paediatric advance care planning as the evidence base is limited therefore studies investigating behavioural aspects of current effective initiation are required. Initiation should be rooted in the knowledge that paediatric advance care planning encompasses wishes, future planning and decision making of the child and family whilst living and should not be focused solely on documenting restrictions to treatment, end of life and funeral plans. Therefore, to ensure families have the time to learn to make decisions and to consider options, initiation of paediatric advance care planning must happen as soon opportune following recognition of a life limiting illness and health professionals must recognise that they hold the key to this happening. Professionals must be aware of the complexities of initiation but must also recognise that these should not act as a barrier to ensuring meaningful conversations occur. The use of a behaviour change theory in further research may provide evidence and on aspects of behaviour which could be adapted or changed to reduce the delay and avoidance behaviour evident in current practice. A standardised approach supported by education, guidelines and clinical tools is required to ensure paediatric advance care planning is initiated as a process and not seen as an anxiety evoking ‘one time’ conversation.

## Conclusion

This review found a dearth of evidence specifically focusing on the initiation of paediatric advance care planning. Overall evidence suggests that health professionals recognise early initiation to be the ideal, and they play a key role ensuring this. Yet ambiguity regarding prognosis, parents’ reactions, who leads, and the skills needed to engage in such conversations act as deterrents in initiating paediatric advance care planning in clinical practice. Consequently, advance care planning conversation occur too late without time for the child and parent to reflect and enact their goals or wishes. Further research is needed on the experience of the initiation process from the professional, parent and child perspective to enable strategies to be developed to ensure conversations occur earlier and are of benefit to all. The identification of behavioural factors impacting on initiation of paediatric advance care planning may inform the development of interventions and to ensure the focus is on the appropriate changeable aspects. Evidence is required, perhaps through the use of a behaviour change theory such as capability, opportunity and motivation theory (COM-B)^71^ in further research to provide evidence on aspects of behaviour which could be adapted or changed to reduce the delay and avoidance behaviour evident in current practice and to ultimately make initiation work for everyone.

## Appendix

**Appendix 1.** Search terms – Integrative Literature Review October 2019.

Palliative care			
Embase		Medline	
exp palliative nursing/ or exp palliative therapy/		exp Palliative Care/	
hospice care/ or hospice/ or hospice nursing/ or		“Hospice and Palliative Care Nursing”/	
hospice patient/		Terminal Care/	
terminal care/		“end of life”.ti,ab.	
death/ or dying/		palliative.ti,ab.	
palliative.ti,ab.hospice*.ti,ab.terminal.ti,ab.“end of life”.ti,ab.(dying adj3 (care or comfort or relief or strateg* or plan or intervention or pain)).ti,ab.(“symptom control” and (dying or death)).ti,ab.(bereavement adj2 support).ti,ab.		(dying adj3 (care or comfort or relief or strateg* or plan or intervention or pain)).ti,ab.“symptom control”.ti,ab.(bereavement adj2 support).ti,ab.**Cochrane**Palliativeterminal*hospice*“end of life”Dyingbereavement	
PsycInfo		CINAHL	
exp Hospice/ or exp “Death and Dying”/ or exp Palliative Care/ or exp Terminally Ill Patients/hospice*.ti,ab.terminal*.ti,ab.“end of life”.ti,ab.(dying adj3 (care or comfort or relief or strateg* or plan or intervention or pain)).ti,ab.(“symptom control” and (dying or death)).ti,ab.(bereavement adj2 support).ti,ab.palliative.ti,ab.		(MH “Palliative Care”) OR (MH “Hospice and Palliative Nursing”) OR (MH “Terminal Care”) OR (MH “Hospice Care”) TX“end of life”TX palliative OR terminal* OR hospice* OR bereavement	
Web of science		SCOPUS	
TOPIC: (palliative* OR terminal* OR hospice* OR dying OR “end of life” OR bereavement)		palliative OR terminal OR hospice OR dying OR “end of life”	
Children			
Cochrane (1)		Medline	
MeSH descriptor Child explode all treesMeSH descriptor Infant explode all trees(child* or adolescen* or infant*)(teenage* or “young people” or “young person” or (young next adult*))(schoolchildren or “school children”)(pediatr* or paediatr*)(boys or girls or youth or youths)MeSH descriptor Adolescent, this term only		exp Child/Adolescent/exp Infant/(child$ or adolescen$ or infant$).af.(teenage$ or young people or young person or young adult$).af.(schoolchildren or school children).af.(pediatr$ or paediatr$).af.(boys or girls or youth or youths).af.or/106-113	
Ovid Embase		Psyc INFO	
exp child/exp ADOLESCENT/exp preschool child/exp infant/(child$ or adolescen$ or infant$).af.(teenage$ or young people or young person or young adult$).af.(schoolchildren or school children).af.(pediatr$ or paediatr$).af.(boys or girls or youth or youths).af.		(adolescence 13–17 yrs or childhood birth 12 yrs or infancy 2–23 mo or neonatal birth 1 mo or preschool age 2–5 yrs or school age 6–12 yrs).ag.(child* or adolescen*).tw.(child* or adololescen* or infant*).tw.(pediatr* or paediatr*).tw.(boys or girls or youth or youths).tw.	
CINAHL		Central Cochrane 2 (update – compare to box 1)	
(MH “Child+”)(MH “Child”)(MH “Infant+”)		exp CHILD/exp ADOLESCENT/exp CHILD, PRESCHOOL/ or CHILD/	
(MH “Adolescence”)(TI child* or adolescen* or infant*) OR (AB child* or adolescen* or infant*)(TI teenage$ or young people or young person or young adult*) OR (AB teenage$ or young people r young person or young adult*)(TI schoolchildren) OR (AB schoolchildren)		exp INFANT/(child$ or adolescen$ or infant$).af.(teenage$ or young people or young person or young adult$).af.(schoolchildren or school children).af.(pediatr$ or paediatr$).af.(boys or girls or youth or youths).af.	
Advanced Care plan			
Cochrane central		Medline	
(“advance care” next (plan or plans or planning)):ti,ab,kw(advance next (directive* or decision*)):ti,ab,kw(living next will*):ti,ab,kw“right to die”:ti,ab,kw((patient or patients) near/5 (advocat* or advocacy)):ti,ab,kw“power of attorney”:ti,ab,kw((“end of life” or EOL) near/5 (care or discuss* or decision* or plan or plans or planning or preference*)):ti,ab,kw“terminal care”:ti,ab,kw(treatment near/5 (refus* or withhold* or withdraw*)).tw.		exp Advance Care Planning/(advance care adj (plan or plans or planning)).tw.(advance adj (directive* or decision*)).tw.living will*.tw.Right to Die/right to die.tw.Patient Advocacy/((patient or patients) adj5 (advocat* or advocacy)).tw.power of attorney.tw.((end of life or EOL) adj5 (care or discuss* or decision* or plan or plans or planning or preference*)).tw.Terminal Care/terminal care.tw.Treatment Refusal/exp Withholding Treatment/(treatment adj5 (refus* or withhold* or withdraw*)).tw.	
Embase		CINAHL	
Living Will/living will*.tw.(advance care adj (plan or plans or planning)).tw.(advance adj (directive* or decision*)).tw.Right to Die/right to die.tw.Patient Advocacy/((patient or patients) adj5 (advocat* or advocacy)).tw.“Power of Attorney”/power of attorney.tw.Terminal care/((end of life or EOL) adj5 (care or discuss* or decision* or plan or plans or planning or preference*)).tw.		(treatment N5 withdraw*) OR AB (treatment N5 withdraw*)(treatment N5 withhold*) OR AB (treatment N5 withhold*)treatment N5 refus*) OR AB (treatment N5 refus*)(terminal care) OR AB (terminal care)(end of life) OR AB (end of life)(power of attorney) OR AB (power of attorney)(patient* N5 advocat*) OR AB (patient* N5 advocat*)(right to die) OR AB (right to die)(living will*) OR AB (living will*)(advance N1 decision*)(advance N1 decision*)(advance N1 directive*)	
Advanced Care plan			
Cochrane central		Medline	
terminal care.tw.Treatment Refusal/Treatment Withdrawal/(treatment adj2 (refus* or withhold* or withdraw*)).tw.		(advance N1 directive*)(advance care N1 plan)(advance care N1 plan*)(MH “Terminal Care+”)(MH “Patient Advocacy”)(MH “Treatment Refusal”)(MH “Right to Die”)(MH “Advance Directives+”)(MH “Advance Care Planning”)	
Health professional allied health, medical/ or nursing/			
**CINAHL**		Central Cochrane	
(health* or medical) and (profession* or personnel or staff or worker* or manpower or workforce)“Health Personnel+”“Health Manpower+”nurse* or AB nurse* or MW nurse**Specialist**ANP Advanced nurse practitioner*physician* or AB physician* or MW physician*doctor* or AB doctor* or MW doctor**Consultant**Paediatrician**Pediatrician*midwive* or AB midwive* or MW midwive**Midwife*S2 (MH “Nursing Staff, Hospital”) or OR (MH “Nurses+”) or (MH “Nursing Role”) or (MH “Nurse Practitioners+”) OR (MH “Advanced Practice Nurses+”) or TI nurs*		MeSH descriptor: [Nursing Staff] explode all treesMeSH descriptor: [Nursing] explode all treesMedline*nurse/ or exp *paramedical personnel/ or exp*physician/ or *medical personnel	
Ovid Medline		Embase	
exp Nurses/ or exp Nursing Staff/ or exp Perioperative Nursing/ or exp Nursing/ or nurs*.ti.		exp nurse practitioner/ or exp advanced practice nurse/ or exp nurse/ or exp perioperative nursing/ or exp nursing staff/ or exp/nursing or nurs*.ti.	
Decision making			
MeSH		Keyword	
Decision making exp		**Medline**	
		decision making.sh.	
		exp choice behavior/	
		(share$ adj decision adj mak$).ti,ab.	
		(decision adj analys$).mp.	
		**EMBASE**	
		decision making.sh.	
		exp choice behavior/	
		(share$ adj decision adj mak$).ti,ab.	
		(decision adj analys$).mp.	
		**BNI**	
		decis$ and mak$).mp.	
		(decis$ and mak$).mp.	
Conversation			
MeSH		Keyword	
**No MeSH available**		**Keyword –** (initiat* or (Conversation* or communication or fishing questions or trigger or QPL or question prompt list or promoting discussions)).mp. [mp=title, abstract, original title, name of substance word, subject heading word, floating sub-heading word, keyword heading word, protocol supplementary concept word, rare disease supplementary concept word, unique identifier, synonyms]	
**Discussion**			
**MeSH**		Discus*	
No MeSH available		talk converse debate, confer deliberate consider,	
**Terms used when initiation of conversations (CPC) (my term –** *Actuate)*			
Conversation starters	Prompts	Things you can say	Initiation/ing
Start	Trigger/s	Fishing questions	QPL (Question prompt list)(Patients)
Promoting discussions	Introducing	Useful questions	Patient Coaching
**Search strategy for current review 24** ^th^ **October 2019**			
**Ovid Medline (Individualised for use in CINAHL, PsycInfo and Embase)**			
Palliative Care/**or** Hospices/**or** “Hospice and Palliative Care Nursing”/**or** terminal care/ or hospice care/ or resuscitation orders/**or** (“end of life” or palliative or (dying adj3 (care or comfort or relief or strateg* or plan or intervention or pain)) or “symptom control” or (bereavement adj2 support)).mp. [mp=abstract, heading words, title]**and**(child* or adolescent or preschool or infant or teenage* or “young people” or “ young person” or schoolchildren or “school children” or pediatr* or paediatr* or boys or girls or youth*).mp. [mp=abstract, heading words, title]**or** Child/**or** Adolescent/**or** Infant/**or** (child$ or adolescen$ or infant$).af.**or** (teenage$ or young people or young person or young adult$).af.**or** (schoolchildren or school children).af.**or** (pediatr$ or paediatr$).af.**or** (boys or girls or youth or youths).af**and**((advance care adj (plan or plans or planning)) or (advance adj (directive* or decision*)) or living will* or Right to Die or Patient Advocacy or ((patient or patients) adj5 (advocat* or advocacy)) or power of attorney or ((end of life or EOL) adj5 (care or discuss* or decision* or plan or plans or planning or preference*)) or Terminal Care or Treatment Refusal or Withholding Treatment or (treatment adj5 (refus* or withhold* or withdraw*)) or plan* ahead).mp. [mp=title, abstract, original title, name of substance word, subject heading word, floating sub-heading word, keyword heading word, protocol supplementary concept word, rare disease supplementary concept word, unique identifier, synonyms] **or** exp Advance Care Planning**or** Right to Die/**or** Patient Advocacy/**or** Terminal Care/**or** Treatment Refusal/**or** Withholding Treatment/**and**(((health* or medical) and (profession* or personnel or staff or worker* or manpower or workforce)) or “Health Personnel+” or “Health Manpower+” or nurse* or Specialist or ANP or “Advanced nurse practitioner” or physician* or doctor* or Consultant or Paediatrician or Pediatrician or midwife* or midwives).mp. [mp=title, abstract, original title, name of substance word, subject heading word, floating sub-heading word, keyword heading word, protocol supplementary concept word, rare disease supplementary concept word, unique identifier, synonyms]**or** exp Nurses/**or** exp Physicians/**or** exp Health Personnel/**and**Palliative Care/**or** Hospices/**or** “Hospice and Palliative Care Nursing”/**or** terminal care/ or hospice care/ or resuscitation orders/**or** (“end of life” or palliative or (dying adj3 (care or comfort or relief or strateg* or plan or intervention or pain)) or “symptom control” or (bereavement adj2 support)).mp. [mp=title, abstract, original title, name of substance word, subject heading word, floating sub-heading word, keyword heading word, protocol supplementary concept word, rare disease supplementary concept word, unique identifier, synonyms]**and**(initiat* or (Conversation* or communication or fishing questions or trigger or QPL or question prompt list or promoting discussions or decision or choice*)).mp. [mp=title, abstract, original title, name of substance word, subject heading word, floating sub-heading word, keyword heading word, protocol supplementary concept word, rare disease supplementary concept word, unique identifier, synonyms]**or** (decision making or choice* or shared decision making).mp. [mp=abstract, heading words, title]			

**Table:** Original search included parents, but this was excluded from final search.

**Parent**, *Mum, Dad*	
**CINAHL**	**Psycinfo**
(parent$ or famil$ or father$ or mother$ or paternal$ or maternal$ or couple$ or marital$).mp.	1. parents (Psycinfo subject heading – exploded) or parent$
	2. mothers (Psycinfo subject heading – exploded) or surrogate parents (humans) (Psycinfo subject heading – exploded) or mother$
	3. fathers (Psycinfo subject heading – exploded) or father$
	4. professional-family relations
	5. family (Psycinfo subject heading – exploded)
Medline	CINAHL
9. family (MeSH – exploded)	9. parents (MeSH – exploded) or parent$
10. mother$	10. mother$
11. father$	11. father$
12. famil$ or parent$	12. exp professional-family relations (MeSH) or professional-family relations
13. professional-family relations (MeSH) or professional-family relations.	13. family (MeSH – exploded)
	14. 9 or 10 or 11 or 12 or 13

**Appendix 2.** Quality appraisal.

Phase 1 JBI Low = <49% Medium = 50 – 74% High = >75%

**Table:** Qualitative papers (n = 6).

Author	Q1	Q2	Q3	Q4	Q5	Q6	Q7	Q8	Q9	Q10	Score	%	Grade
Henderson^44^	UN	Y	Y	Y	Y	N	N	Y	Y	Y	7/10	70	M
Hiscock and Barclay^45^	N	Y	Y	Y	Y	N	N	Y	UN	Y	6/10	60	M
Jack et al.^46^	Y	Y	Y	Y	Y	N	N	Y	Y	Y	8/10	80	H
Lotz et al.^24^	UN	Y	Y	Y	Y	Y	Y	Y	Y	Y	9/10	90	H
Mitchell and Dale^15^	UN	Y	Y	Y	Y	Y	N	Y	Y	Y	8/10	80	H
Zaal-Schuller et al.^47^	N	Y	Y	Y	Y	Y	N	Y	Y	Y	8/10	80	H

**Table:** Quantiative papers (n = 8).

Author	Q1	Q2	Q3	Q4	Q5	Q6	Q7	Q8	Q9	Score	%	Grade
Basu and Swil^11^	Y	UN	Y	N	UN	Y	Y	UN	Y	5/9	55	M
Bradford et al.^52^ (Delphi)	Y	UN	UN	Y	Y	Y	Y	Y	UN	6/9	66	M
De Vos et al.^48^	Y	Y	Y	UN	Y	Y	Y	Y	Y	8/9	88	H
Durall et al.^27^	Y	Y	UN	UN	Y	Y	Y	Y	Y	7/9	77	H
Forbes et al.^49^	Y	Y	Y	Y	Y	UN	UN	UN	Y	6/9	66	M
Harrison et al.^50^	N	UN	UN	N	Y	N	N	Y	UN	2/9	22	L
Kruse et al.^51^	Y	Y	Y	Y	Y	N	Y	Y	Y	8/9	88	H
Sanderson et al.^25^	y	UN	UN	Y	y	N	Y	y	UN	5/9	55	M

**Table:** Non-research (n = 7).

Author	Q1	Q2	Q3	Q4	Q5	Q6	Score	%	Grade
Harrop et al.^53^	Y	UN	Y	Y	Y	Y	5/6	83	H
Haynes et al.^54^	Y	Y	Y	Y	N	NA	4/6	66	M
Mack & Joffe^55^	Y	UN	Y	Y	Y	Y	5/6	83	H
Pao & Mahoney^56^	UN	UN	Y	Y	Y	Y	4/6	66	M
Sidgwick et al.^57^	Y	Y	Y	Y	Y	Y	6/6	100	H
Tasi^58^	Y	Y	Y	Y	Y	Y	6/6	100	H
Wiener et al.^59^	Y	UN	Y	Y	Y	Y	5/6	83	H

**Appendix 3.** Data on retained papers.

Country	Amount	Reference (number)
Australia	4	Basu and Swil^11^; Henderson^44^; Forbes et al.^49^
Canada	1	Tasi^58^
Germany	1	Lotz et al.^24^
Netherlands	2	Zaal-Schuller et al.^47^; De Vos et al.^48^
UK	6	Mitchell and Dale^15^; Hiscock and Barclay^45^; Jack et al.^46^; Harrop et al.^53^; Haynes et al.^54^; Sidgwick et al.^57^
USA	7	Sanderson et al.^25^; Durall et al.^27^; Harrison et al.^50^; Kruse et al.^51^; Mack and Joffe^55^; Pao and Mahoney^56^; Wiener et al.^59^
**Study design**		
Qualitative	6	Mitchell and Dale^15^; Lotz et al.^24^; Henderson^44^; Hiscock and Barclay^45^; Jack et al.^46^; Zaal-Schuller et al.^47^
Quantitative inc. Delphi	8	Basu and Swil^11^; Sanderson et al.^25^; Durall et al.^27^; De Vos et al.^48^; Forbes et al.^49^; Harrison et al.^50^; Kruse et al.^51^; Bradford et al.^52^
Theoretical	7	Harrop et al.^53^; Haynes et al.^54^; Mack and Joffe^55^; Pao and Mahoney^56^; Sidgwick et al.^57^; Tasi^58^; Wiener et al.^59^
**Published year**		
2008–2010	2	Forbes et al.^49^; Tasi^58^
2011–2013	4	Sanderson et al.^25^; Durall et al.^27^; De Vos et al.^48^; Wiener et al.^59^
2014–2016	6	Mitchell and Dale^15^; Lotz et al.^24^; Zaal-Schuller et al.^47^; Harrison et al.^50^; Bradford et al.^52^; Mack and Joffe^55^
2017–2019	9	Basu and Swil^11^; Henderson^44^; Hiscock and Barclay^45^; Jack et al.^46^; Kruse et al.^51^; Harrop et al.^53^; Haynes et al.^54^; Pao and Mahoney^56^; Sidgwick et al.^57^
**Sample**		
Doctor	12	Basu and Swil^11^; Mitchell and Dale^15^; Lotz et al.^24^; Sanderson et al.^25^; Hiscock and Barclay^45^; Jack et al.^46^; Zaal-Schuller et al.^47^; De Vos et al.^48^; Forbes et al.^49^; Harrison et al.^50^; Kruse et al.^51^; Bradford et al.^52^
Trainee/Junior Doctor	3	Basu and Swil^11^; Forbes et al.^49^; Kruse et al.^51^
Nurse	10	Mitchell and Dale^15^; Lotz et al.^24^; Sanderson et al.^25^; Durall et al.^27^; Henderson^44^; Hiscock and Barclay^45^; Jack et al.^46^; Harrison et al.^50^; Kruse et al.^51^; Bradford et al.^52^
Senior/Advance practice Nurse	6	Mitchell and Dale^15^; Sanderson et al.^25^; Durall et al.^27^; Henderson^44^; Jack et al.^46^; Kruse et al.^51^
Allied Health Professional/psychosocial	6	Lotz et al.^24^; Henderson^44^; Hiscock and Barclay^45^; Jack et al.^46^; Harrison et al.^50^; Bradford et al.^52^
Parents	1	Zaal-Schuller et al.^47^
Midwives	1	Jack et al.^46^
Care Assistant	1	Jack et al.^46^
Bereavement support	1	Jack et al.^46^
N/A	7	Harrop et al.^53^; Haynes et al.^54^; Mack and Joffe^55^; Pao and Mahoney^56^; Sidgwick et al.^57^; Tasi^58^; Wiener et al.^59^
**Setting**		
PICU/ICU/CICU	6	Mitchell and Dale^15^; Sanderson et al.^25^; Durall et al.^27^; De Vos et al.^48^; Kruse et al.^51^; Sidgwick et al.^57^
Haem/ Oncology wards	4	Sanderson et al.^25^; Durall et al.^27^; De Vos et al.^48^; Kruse et al.^51^
Neurology/ Metabolic	2	Hiscock and Barclay^45^; De Vos et al.^48^
Whole Children’s Hospital or no ward specified.	6	Basu and Swil^11^; Lotz et al.^24^; Jack et al.^46^; Forbes et al.^49^; Harrison et al.^50^; Haynes et al.^54^
Children’s Hospice	1	Jack et al.^46^
Community	1	Jack et al.^46^
Care facility	2	Lotz et al.^24^
Out patient Department	3	Lotz et al.^24^; Sanderson et al.^25^; Durall et al.^27^
Neonatal	1	Jack et al.^46^
N/A	3	Henderson^44^; Zaal-Schuller et al.^47^; Bradford et al.^52^
